# The Diagnostic Value of Mitochondrial Mass of Peripheral T Lymphocytes in Early Sepsis

**DOI:** 10.3389/fpubh.2022.928306

**Published:** 2022-07-14

**Authors:** Ling-Xiao Pang, Wen-Wei Cai, Lue Chen, Jin Fu, Chun-Xiao Xia, Jia-Yan Li, Qian Li

**Affiliations:** ^1^Emergency and Critical Care Center, Department of Emergency Medicine, Zhejiang Provincial People's Hospital (Affiliated People's Hospital, Hangzhou Medical College), Hangzhou, China; ^2^Graduate School of Clinical Medicine, Bengbu Medical College, Bengbu, China; ^3^Zhejiang Chinese Medical University, Hangzhou, China

**Keywords:** mitochondrial mass, T lymphocytes, sepsis, receiver operating characteristic curve (ROC), mitochondrial function

## Abstract

**Background:**

Studies have shown that lymphocyte dysfunction can occur during the early stages of sepsis and that cell dysfunction is associated with mitochondrial dysfunction. Therefore, quantifying the mitochondrial function of lymphocytes in patients with sepsis could be valuable for the early diagnosis of sepsis.

**Methods:**

Seventy-nine patients hospitalized from September 2020 to September 2021 with Sepsis-3 were retrospectively analyzed and subsequently compared with those without sepsis.

**Results:**

Univariate analysis showed statistical differences between the data of the two groups regarding age, neutrophil/lymphocyte, procalcitonin (PCT), C-reactive protein, total bilirubin, serum creatinine, type B natriuretic peptide, albumin, prothrombin time, activated partial thromboplastin time, lactic acid, single-cell mitochondrial mass (SCMM)-CD3, SCMM-CD4, SCMM-CD8, and Acute Physiology and Chronic Health Evaluation II score (*P* < 0.05). Multivariate logistic regression analysis performed on the indicators mentioned above demonstrated a statistical difference in PCT, lactic acid, SCMM-CD4, and SCMM-CD8 levels between the two groups (*P* < 0.05). The receiver operating characteristic curves of five models were subsequently compared [area under the curve: 0.740 (PCT) vs. 0.933 (SCMM-CD4) vs. 0.881 (SCMM-CD8) vs. 0.961 (PCT + SCMM-CD4) vs. 0.915 (PCT+SCMM-CD8), *P* < 0.001].

**Conclusion:**

SCMM-CD4 was shown to be a better diagnostic biomarker of early sepsis when compared with the traditional biomarker, PCT. Furthermore, the value of the combination of PCT and SCMM-CD4 in the diagnosis of early sepsis was better than that of SCMM-CD4 alone.

## Introduction

Sepsis is a systemic inflammatory response syndrome caused by a severe infection. Previous research has shown that the pathophysiological process of sepsis is essentially a process of immune system disorders. Despite the hyperimmune responses witnessed during the initial stage of sepsis, the state progresses to immunosuppression (1), resulting in secondary or recurrent infections. In the long run, patients' conditions will deteriorate due to repetitive infection, ultimately developing multiple organ dysfunction syndromes (2). Studies have established that immune paralysis caused by sepsis is associated with a decrease in the number of immune cells and immune system disorders (3). In contrast, cell dysfunction is associated with mitochondrial function damage (4). The human body relies on mitochondria for metabolism and energy conversion, indicating that once mitochondrial function becomes impaired, it seriously affects cell operation and eventually leads to tissue and organ damage. Recently, a new immunofluorescence technology that reflects mitochondrial function has emerged, detecting mitochondrial quality and expressing it using single-cell mitochondrial mass (SCMM) (5). Therefore, this study discusses the differences in SCMM of T lymphocytes between patients with and without sepsis and evaluates the value of SCMM of T lymphocytes in the diagnosis of sepsis.

## Materials and Methods

### Study Population

Seventy-nine patients who were hospitalized in the emergency intensive care unit of Zhejiang Provincial People's Hospital from September 2020 to September 2021, diagnosed with Sepsis-3 and infection, using a quick sequential organ failure assessment score of ≥2 (6), were included in this study. The exclusion criteria were as follows: (i) patients aged <18 years; (ii) patients with a history of malignant tumor; (iii) patients who underwent organ transplantation or were long-term users of drugs that affect immune function (such as adrenocortical hormone); (iv) patients with renal failure requiring kidney replacement therapy; and (v) patients whose hospital stay was less than 48 h. Blood, urine, and sputum cultures were performed on all patients, coupled with ultrasound and computed tomography to determine the location of infection. Evidence of infection refers to the presence of focal infections or results of bacteriological examination. Seventy-five patients without sepsis, hospitalized during the same period as those with sepsis, were selected for comparison. The Ethics Committee of the Zhejiang Provincial People's Hospital (the People's Hospital of Hangzhou Medical College) approved the research protocol of this study. Informed consent was obtained from patients or their families for all treatments and indicators obtained.

### Flow Cytometry

**Figure 1:**
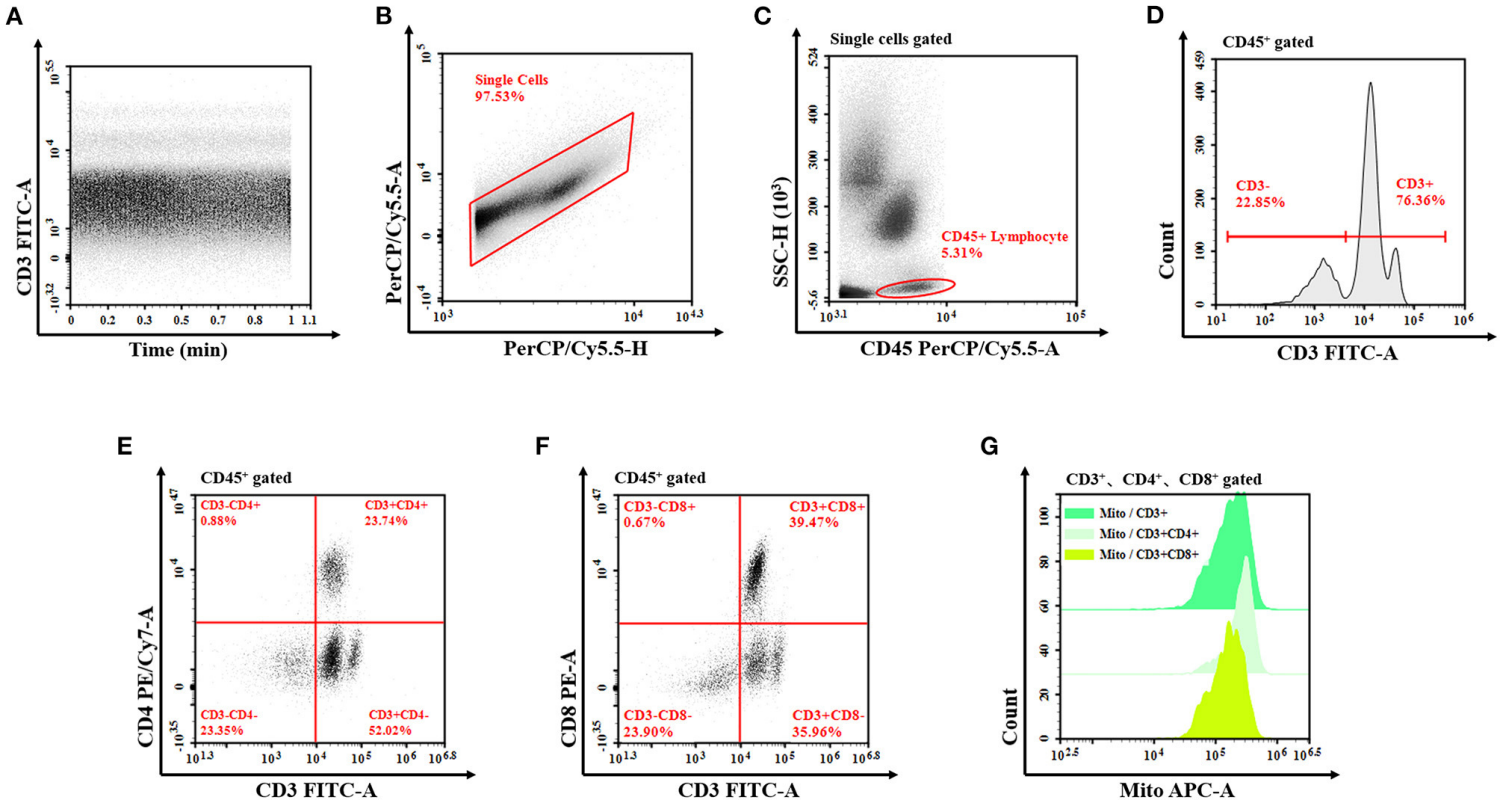
Flow cytometry of T lymphocyte mitochondrial mass. **(A)** The analysis is started by examining the event count over time in order to exclude irregularities from the analysis. **(B)** Exclude the doublets from further analysis by investigating the PerCP/Cy5.5 signal for both the peak and the TOF characteristics compared to the integral attributes. **(C)** The remaining events are analyzed for their CD45 vs. SSC characteristics to define the lymphocyte after single-cell signal acquisition. Draw a gate around the cell population (all lymphocyte cells gate). **(D–F)** In the next step, the T cells (CD3+ T cells, CD3+CD4+ T cells, and CD3+CD8+ T cells) are determined due to the expression of CD3, CD4, and CD8, then set the corresponding gate, respectively. **(G)** Show these fluorescence characteristics of mitochondria by the half-offset histogram of the APC channel. The mitochondrial mass is detected by the MFI of APC. TOF, time of flight; APC, allophycocyanin; MFI, median fluorescence index.

Data on indicators, such as population characteristics, infection markers, organ function, Acute Physiology and Chronic Health Evaluation (APACHE) II score, and flow cytometry data of patients with and without sepsis were collected, and univariate and multivariate analyses were performed. Peripheral venous blood was collected 48 h after the diagnosis was established, into collection tubes coated with EDTA-k anticoagulant and examined using flow cytometry. Among the indicators assessed were percentage and absolute counts of lymphocytes, T cells, and their subsets of helper T cells (Th cells: CD3 ^+^ CD4 ^+^ CD8^−^), killer T cells (TS cells: CD3 ^+^ CD4^−^ CD8 ^+^), and subsets of mitochondrial mass (MM) and SCMM. MM was measured using the medium fluorescence index of mitochondria (5, 7). SCMM of T lymphocytes was obtained by calculating the absolute count of MM and cells (Figure 1). Flow cytometry was performed using NovoCyte (Agilent Technologies, US), and flow cytometry antibodies CD3/CD8/CD4/CD45 and Mito dye were produced by UBBIO LTD (Zhejiang, China).

### Statistical Analysis

The data were processed using Statistical Package for Social Sciences v. 25.0. Normal distribution statistics are expressed as mean ± SD, and univariate comparisons between sepsis and non-sepsis groups were performed using the independent samples *t*-test. Measurements of skewed distribution are expressed as medians and quartiles, and the rank-sum test was used for univariate comparison between groups. Count data of the two groups were compared using the χ^2^ test. Multivariate logistic regression analysis was performed to control for confounding bias and explore the risk factors affecting the prognosis of sepsis. The receiver operating characteristic (ROC) curves were compared using MedClac. *P* < 0.05 was considered statistically significant for all of the indicators, as mentioned earlier.

## Results

### Univariate Analysis of Clinical Data Between the Two Groups

This study included 79 and 75 patients in the sepsis and non-sepsis groups, respectively. There were 52 and 40 males in the sepsis (65.8%) and non-sepsis (53.3%) groups, respectively. Univariate analysis was performed for sex, age, body temperature, neutrophil/lymphocyte (N/L), procalcitonin (PCT), white blood cell, C-reactive protein (CRP), total bilirubin (TB), glutamic-pyruvic transaminase, serum creatinine (Scr), type B natriuretic peptide (BNP), mean artery pressure, oxygenation index, pH, albumin (Alb), prothrombin time (PT), activated partial thromboplastin time (APTT), lactic acid (Lac), SCMM-CD3, SCMM-CD4, SCMM-CD8, duration of hospital stay, APACHE II score, number of underlying medical conditions, and emergency surgery between the two groups. The results showed that there were significant differences between the two groups in terms of age, N/L, PCT, CRP, TB, Scr, BNP, Alb, PT, APTT, Lac, SCMM-CD3, SCMM-CD4, SCMM-CD8, and APACHE II score (Table 1).

**Table 1:** Univariate analysis of the variables of the groups with and without sepsis.

**Variable**	**Sepsis (*n* = 79)**	**No sepsis (*n* = 75)**	**χ^2^/T/U test**	
			**χ^2^/T/Z**	***P*-value**
**Sex**				
Male *N* (%)	52 (65.80)	40 (53.30)	2.50	0.114
Female *N* (%)	27 (34.20)	35 (46.70)		
Age (years)	69.91 ± 14.83	64.24 ± 16.18	2.27	0.025^*^
Temp (°C)	37.40 ± 0.94	37.32 ± 0.84	0.55	0.585
N/L (%)	15.70 (11.00, 28.20)	8.30 (5.30, 17.80)	−4.56	<0.001^***^
PCT (ng/mL)	4.60 (0.53, 18.00)	0.54 (0.11, 1.80)	−5.14	<0.001^***^
WBC (10^9^/L)	10.20 (7.14, 16.29)	10.57 (7.10, 14.44)	−0.33	0.744
CRP (mg/L)	96.80 (47.50, 214.50)	54.20 (19.50, 105.20)	−3.78	<0.001^***^
TB (μmol/L)	17.10 (12.20, 37.00)	13.80 (9.10, 20.80)	−3.59	<0.001^***^
GPT (U/L)	26.00 (16.00, 61.00)	30.00 (13.00, 52.00)	−0.31	0.759
Scr (μmol/L)	104.40 (71.10, 175.00)	73.10 (62.80, 104.80)	−3.08	0.002^**^
BNP (pg/ml)	368.60 (143.50, 1371.60)	126.75 (65.63, 309.88)	−4.55	<0.001^***^
MAP (mmHg)	84.09 ± 15.86	86.65 ± 14.04	−1.06	0.293
OI (mmHg)	278.04 ± 121.13	311.31 ± 129.97	−1.64	0.102
PH	7.40 ± 0.78	7.42 ± 0.06	−1.64	0.104
Alb (g/L)	28.90 ± 4.27	31.27 ± 4.12	−3.51	0.001^**^
PT (s)	14.20 (12.90, 17.90)	13.10 (12.30, 14.10)	−3.22	0.001^**^
APTT (s)	32.60 (27.40, 39.00)	27.70 (25.60, 30.80)	−3.46	0.001^**^
Lac (mmol/L)	2.30 (1.50, 3.60)	1.50 (1.00, 2.30)	−4.27	<0.001^***^
SCMM-CD3	156.00 (94.07, 266.61)	35.19 (17.74, 68.92)	−9.20	<0.001^***^
SCMM-CD4	343.73 (179.44, 576.94)	68.23 (35.77, 130.21)	−9.27	<0.001^***^
SCMM-CD8	424.28 (204.38, 673.91)	97.73 (38.84, 175.92)	−8.15	<0.001^***^
Hospital day	7 (5, 14)	10 (6, 21)	−1.93	0.054
APACHE II score	21.99 ± 7.45	18.45 ± 7.03	3.02	0.003^**^
Number of basic diseases	2 (1, 3)	1 (0, 2)	−1.78	0.075
Emergency surgery (%)	19 (24.1%)	20 (26.7%)	0.14	0.709

*Data are shown as the mean ± SD and M (IQR)*.

* *, P <0.05*;

** *, P <0.01*;

*** *, P <0.001*.

*TEMP, temperature; N/L, neutrophil/lymphocyte; PCT, procalcitonin; WBC, white blood cell; CRP, C-reactive protein; TB, total bilirubin; GPT, glutamic-pyruvic transaminase; Scr, serum creatinine; BNP, type B natriuretic peptide; MAP, mean arterial pressure; OI, oxygenation index; Alb, albumin; PT, prothrombin time; APTT, activated partial thromboplastin time; Lac, lactic acid; SCMM, single-cell mitochondrial mass; SD, standard deviation; M, median; IQR, interquartile range*.

### Multivariate Logistic Regression Analysis of the Significant Indices

Following univariate analysis of age, N/L, PCT, CRP, TB, Scr, BNP, Alb, PT, APTT, Lac, SCMM-CD4, SCMM-CD8, and APACHE II score in both groups, multivariate logistic regression analysis was performed on indicators that showed statistical differences to eliminate the interference of confounding factors. Statistical differences were observed in the PCT, Lac, SCMM-CD4, and SCMM-CD8 between the two groups (Table 2).

**Table 2:** Multivariate logistic regression of the significant variables in the groups with and without sepsis.

**Variable**	**B**	**S.E**.	**Wald**	**OR (95% Cl)**	***P*-value**
Age	0.000	0.024	0.000	1.000 (0.954–1.048)	0.995
N/L	−0.005	0.045	0.015	0.995 (0.910–1.087)	0.903
PCT	0.144	0.068	4.505	1.155 (1.011–1.319)	0.034^*^
CRP	0.005	0.005	1.080	1.005 (0.995–1.015)	0.299
TB	−0.013	0.024	0.281	0.987 (0.941–1.036)	0.596
Scr	−0.003	0.005	0.289	0.997 (0.988–1.007)	0.591
BNP	0.000	0.000	0.210	1.000 (0.999–1.001)	0.647
Alb	−0.039	0.082	0.229	0.962 (0.819–1.129)	0.633
PT	−0.102	0.091	1.251	0.903 (0.755–1.080)	0.263
APTT	0.013	0.036	0.136	1.013 (0.944–1.088)	0.712
Lac	0.498	0.228	4.789	1.646 (1.053–2.571)	0.029*
SCMM-CD4	0.026	0.007	15.485	1.026 (1.013–1.040)	<0.001^***^
SCMM-CD8	0.009	0.004	5.366	1.009 (1.001–1.017)	0.021*
Hospital day	0.003	0.017	0.025	1.003 (0.971–1.036)	0.874
APACHE II score	−0.022	0.064	0.115	0.978 (0.863–1.110)	0.734

*B, Co-efficient for the constant in the null model; S.E., standard error around the Co-efficient*.

*For the constant; Wald, Wald chi-square test value; OR, odds ratio; CI, confidence interval*.

* *P <0.05*;

*** *P <0.001; N/L, neutrophil/lymphocyte; PCT, procalcitonin; CRP, C-reactive protein; TB, total bilirubin; Scr, serum creatinine; BNP, type B natriuretic peptide; Alb, albumin; PT, prothrombin time; APTT, activated partial thromboplastin time; Lac, lactic acid; SCMM, single-cell mitochondrial mass*.

### Predictive Efficacy of PCT, SCMM-CD4, and SCMM-CD8

**Figure 2:**
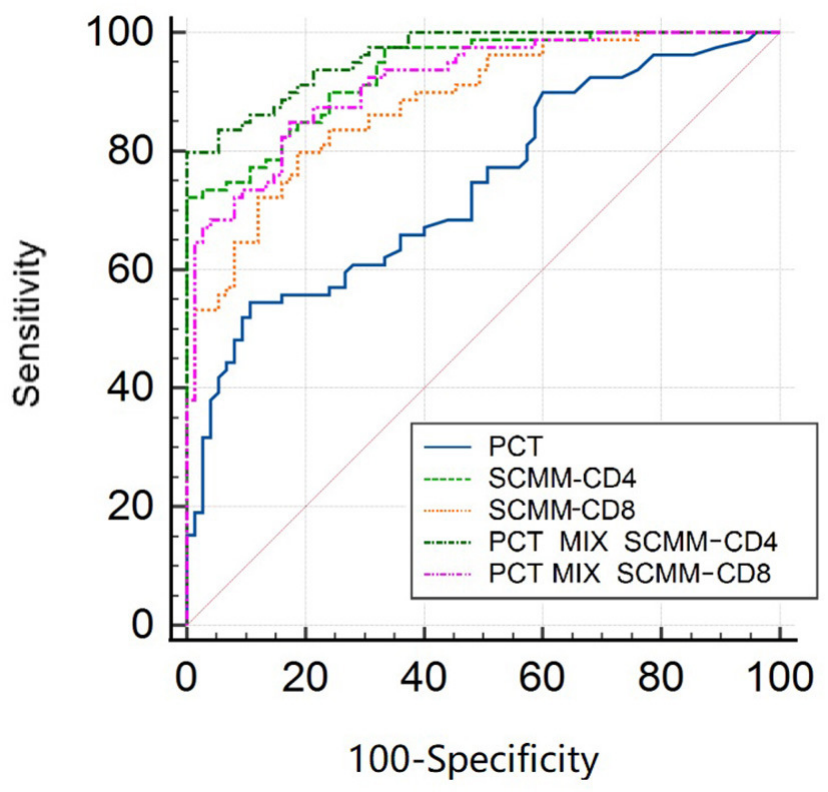
ROC curve of sepsis detected by SCMM-CD4, SCMM-CD8, and their combination with PCT. ROC, receiver operating characteristic; SCMM, single-cell mitochondrial mass; PCT, procalcitonin.

The area under the ROC curve (AUC) was used to evaluate the predictive efficacy of PCT, SCMM-CD4, and SCMM-CD8 levels in sepsis. The results showed that when the PCT cut-off value was 3.66 ng/ml, the AUC to distinguish patients with sepsis from those without sepsis was 0.740 (sensitivity 54.4%, specificity 89.3%, and Youden index 0.437). When the SCMM-CD4 cut-off value was 229.92, the AUC for distinguishing patients with sepsis from those without sepsis was 0.933, with a sensitivity of 72.2%, specificity of 98.7%, and Youden index of 0.722. When the SCMM-CD8 cut-off value was 188.44, the AUC for distinguishing patients with sepsis from those without sepsis was 0.881, with a sensitivity of 79.7%, specificity of 81.3%, and Youden index of 0.611. Furthermore, we combined SCMM-CD4 and SCMM-CD8 with PCT, respectively, to see whether the prediction model could be more optimized or not. The results demonstrated that when the PCT+SCMM-CD4 cut-off value was 0.71, the AUC for distinguishing patients with sepsis from those without sepsis was 0.961, with a sensitivity of 79.7%, specificity of 99.3%, and Youden index of 0.798. When the PCT+SCMM-CD8 cut-off value was 0.40, the AUC for distinguishing patients with sepsis from those without sepsis was 0.915, with a sensitivity of 84.8%, specificity of 82.7%, and Youden index of 0.675. The ROC curves of the five models, which were compared, differed significantly except for the SCMM-CD4 vs. PCT+SCMM-CD8 model (Figure 2 and Tables 3, 4).

**Table 3:** Diagnostic values of SCMM-CD4, SCMM-CD8, and their combination with PCT in sepsis.

**Variable**	**AUC**	**S.E**.	**Sig**.	**95% CI**
PCT	0.740	0.040	<0.001^***^	0.662–0.817
SCMM-CD4	0.933	0.018	<0.001^***^	0.897–0.969
SCMM-CD8	0.881	0.026	<0.001^***^	0.819–0.927
PCT+SCMM-CD4	0.961	0.013	<0.001^***^	0.936–0.986
PCT+SCMM-CD8	0.915	0.022	<0.001^***^	0.859–0.954

*AUC, area under the curve; S.E., standard error around the Co-efficient for the constant; CI, confidence interval*.

*** *P <0.001; PCT, procalcitonin; SCMM, single-cell mitochondrial mass*.

**Table 4:** Comparison between the ROC curves of five models.

**Variable**	**AUC difference**	**S.E**.	**Z**	**95% CI**	**Sig**.
PCT vs. SCMM-CD4	0.193	0.045	4.29	0.105–0.281	<0.001^***^
PCT vs. SCMM-CD8	0.141	0.048	2.96	0.048–0.023	0.003^**^
PCT vs. PCT+SCMM-CD4	0.021	0.039	5.71	0.145–0.297	<0.001^***^
PCT vs. PCT+SCMM-CD8	0.175	0.038	4.63	0.101–0.249	<0.001^***^
SCMM-CD4 vs. SCMM-CD8	0.052	0.024	2.13	0.004–0.100	0.032^*^
SCMM-CD4 vs. PCT+SCMM-CD4	0.028	0.013	2.16	0.003–0.054	0.030^*^
SCMM-CD4 vs. PCT+SCMM-CD8	0.018	0.025	0.711	−0.031–0.067	0.478
SCMM-CD8 vs. PCT+SCMM-CD4	0.080	0.026	3.050	0.029–0.132	0.002^**^
SCMM-CD8 vs. PCT+SCMM-CD8	0.034	0.017	1.995	0.001–0.068	0.046^*^
PCT+SCMM-CD4 vs. PCT+SCMM-CD8	0.046	0.021	2.231	0.006–0.087	0.026^*^

*AUC, area under the curve; S.E., standard error around the Co-efficient for the constant; CI, confidence interval*.

* *P <0.05*;

** *P <0.01*;

*** *P <0.001; PCT, procalcitonin; SCMM, single-cell mitochondrial mass*.

## Discussion

The results of the present study revealed statistical differences in age, N/L, PCT, CRP, TB, Scr, BNP, Alb, PT, APTT, Lac, SCMM-CD3, SCMM-CD4, SCMM-CD8, and APACHE II score between the two groups (Table 1). However, since the present study adopted a case-control approach with an uneven distribution, multivariate logistic regression analysis was performed for statistically different indicators to eliminate the interference of confounding factors. Because SCMM-CD3 had the problem of multicollinearity in multivariate logistic regression, we excluded this indicator in multivariate logistic regression analysis. The results showed that only PCT, Lac, SCMM-CD4, and SCMM-CD8 levels had statistically significant differences (Table 2). Because Lac is not a specific indicator of sepsis, we did not determine its predictive value for early sepsis. After drawing ROC curves for PCT, SCMM-CD4, and SCMM-CD8, the AUC of SCMM-CD4 was the best of the three indicators, suggesting that SCMM-CD4 had a higher value in the early prediction of sepsis.

Studies have shown that sepsis causes a decline in lymphocyte counts and function (8). Although some scholars believe that immunosuppression caused by sepsis occurs during the later stages of sepsis (2), research has shown that the number and function of lymphocytes can decrease 48 h after the diagnosis of sepsis. For example, Inoue et al. showed that 48 h after the diagnosis of sepsis, the CD28 lymphocyte subsets of CD4 lymphocytes decreased. At the same time, the percentage of immunosuppressive PD-1 ^+^ T cells and regulatory T cells increases among geriatric patients with sepsis (9). Cabrera-Perez et al. (10) established that on the second day after mice experienced sepsis impairment, the total number of CD4 lymphocytes in the spleen, inguinal lymph nodes, and blood decreased significantly, along with a decrease in interleukin-17. Therefore, the study population selected for the present study included patients with sepsis hospitalized for more than 48 h. Lymphocytes can play an anti-inflammatory role and repress inflammation by secreting cytokines. In sepsis, changes in the mRNA levels of T-bet, GATA3, and ROR-γT result in the differentiation of Th1, Th2, and Th17 lymphocyte subsets and subsequent changes in secreted inflammatory factors (11–13). CD4 + cells in patients with sepsis have an increased expression of inhibitory receptors, including PD-1, 2B4, BTLA, and TRAIL, which could lead to a weakened immune response (14–17).

In addition to the mechanisms mentioned above, lymphocyte function is also closely related to mitochondrial function. One of the mechanisms of mitochondrial function damage is damage to mitochondrial protein turnover and regeneration (18). Since reactive oxygen species (ROS) are constantly produced during the oxidative phosphorylation of mitochondria, organelles are vulnerable to DNA mutations or protein misfolding (19). Therefore, a quality control system is required to ensure protein functionality. A recent study found that most mitochondrial protein turnover (~70%) occurs through a variety of non-autophagic degradation processes, such as mitochondrial proteases, the ubiquitin-proteasome system (UPS), and mitochondrial-derived vesicles (MDV). Mild mitochondrial damage is addressed by activating specific proteases in each mitochondrial compartment to degrade misfolded or oxidized proteins (20). The deubiquitinase USP30 (21), E3 ubiquitin ligase Parkin (22), mitochondrial ubiquitin ligases MARCHV/MITOL (23), MAPL/MULAN (24), and RNF185 (25) are located in the outer mitochondrial membrane to mediate protein polyubiquitination. These UPS components can remove damaged proteins and regulate mitochondrial morphology and renewal. Mitochondria also undergo dynamic remodeling through repeated fusion and division of the mitochondrial membrane, which can redistribute energy in the mitochondrial potential, metabolites, proteins, and mitochondrial DNA (26, 27) to avoid the accumulation of dysfunctional mitochondria and to maintain their overall function (28). MDV transfer misfolded or oxidized proteins and lipids in the mitochondria to lysosomes for degradation (29). These findings indicate that mitochondria must undergo dynamic renewal to maintain normal cellular function. Diseases lead to mitochondrial dysfunction, preventing the removal of aging mitochondria and further affecting cell function. Therefore, finding a convenient and rapid detection index for the quality of cell mitochondria is of great significance for evaluating the role of immune cells in sepsis, and the emergence of SCMM of T lymphocytes meets this clinical demand.

A fluorescent probe, MitoTracker, is used to detect MM by fluorescently labeling the mitochondria of lymphocytes to reflect the quality of the mitochondria (7). A similar research study, conducted by Doherty et al. (30) established that detection of ROS markers (HE and DHR) and reactive nitrogen species markers (DCF-DA and DAF-FM) using MitoTracker, was significantly correlated with the production of ROS and nitrosative stress in the cytoplasm and mitochondria. Yu et al. (5) found that HIV infection leads to an increase in MM in CD4^+^T and CD8^+^T cells, resulting in the accumulation of ROS in CD4^+^T cells, affecting their function. SCMM uses Mito tracker's fluorescent probe to detect the mitochondrial fluorescence intensity of lymphocyte subsets by flow cytometry and then divides it by the count of corresponding lymphocyte subsets to obtain the SCMM of each lymphocyte subset. Compared to MM, SCMM can more sensitively reflect the function of cell mitochondria, which is an innovation in the detection of cell mitochondrial function. The higher SCMM in peripheral blood lymphocytes reflects abnormal mitochondrial metabolism, which is positively correlated with the degree of mitochondrial damage. The present study showed that there was a statistical difference in SCMM-CD4 and SCMM-CD8 between the groups with and without sepsis, which also confirms the argument that sepsis affects the function of lymphocytes by damaging lymphocyte mitochondria. Since this analytical method was more sensitive to CD4 lymphocytes than CD8 ones (7), the predictive value of SCMM-CD8 was not as high as that of SCMM-CD4, which was also consistent with the AUC results of our study. As CD3 includes CD4 and CD8, there was a collinearity problem; therefore, it was not used as a parameter of multivariate logistic regression. However, this research was only able to reflect the overall situation of mitochondrial quality through SCMM of T lymphocytes without clarifying which kind of mitochondrial regeneration mechanism was damaged.

After drawing ROC curves for PCT, SCMM-CD4, SCMM-CD8, PCT+SCMM-CD4, and PCT+SCMM-CD8, the present study found that the AUC of SCMM-CD4 or SCMM-CD8 was greater than that of PCT. PCT is a widely used classic indicator in clinical practice and is mainly used to reflect the severity of gram-negative infections (31). It is often used as a reference for the diagnostic efficacy of new biomarkers of sepsis (32). The present study found that when comparing PCT with SCMM-CD4 or SCMM-CD8 cut-off values, the sensitivity and specificity of SCMM-CD4 or SCMM-CD8 were higher than those of PCT, indicating that the diagnostic value of SCMM-CD4 or SCMM-CD8 in the early prediction of sepsis is higher than that of PCT. Moreover, SCMM-CD4 was better than SCMM-CD8. We further studied the predictive value of SCMM-CD4 or SCMM-CD8 combined with PCT for early sepsis, and the results showed that SCMM-CD4 combined with PCT had the best predictive effect (Figure 2 and Tables 3, 4). Some studies have shown that mitochondrial function is related to patient prognosis. A meta-analysis by Wang et al. (33) showed that mitochondrial metabolic indices could predict the mortality of patients with sepsis. Maestraggi et al. (34) established that abnormal mitochondrial function of skeletal muscle and lymphocytes during septic shock could trigger intensive care unit-acquired weakness, infectious, and immune paralysis. Therefore, based on the mechanism of mitochondrial damage in sepsis, selecting an appropriate target intervention may be an effective measure to reverse the process of sepsis and improve its prognosis in the future (35). Currently, 5-hydroxydecanoate can block the particle K_ATP_ channel, thereby preventing an increase in line permeability and ATP outflow (36). Mitochondria-targeted Co-enzyme Q10 can target and aggregate into mitochondria to improve the electron transport chain function (37). Animal experiments have confirmed that recombinant human mitochondrial transcription factor A stimulates mitochondrial regeneration, which can significantly increase the expression of mitochondrial DNA and improve mitochondrial function (38). The present study has several limitations, such as the limited sample size, the effect of blood collection time, disease onset, and other factors. Therefore, bias may have existed in the research results. In addition, the present study neither clarified whether SCMM-CD4 could be used as an indicator to judge the prognosis of sepsis, nor did it examine downstream indicators reflecting abnormal mitochondrial protein turnover and regeneration. These issues need to be studied further by collecting more sample data.

The present study established that differences exist in lymphocyte mitochondrial functions between patients with sepsis and those without sepsis. SCMM-CD4 and SCMM-CD8, which reflect lymphocyte mitochondrial function, have better predictive value for early sepsis than PCT, a classical sepsis biomarker. SCMM-CD4 combined with PCT has the highest predictive value. Although the detection method of SCMM in T lymphocytes cannot clarify the specific mechanism of mitochondrial regeneration disorder leading to its functional damage, it can still reflect the overall level of mitochondrial function in patients. Because SCMM of T lymphocytes is superior to PCT in the diagnosis of early sepsis, it will assist in the early identification and treatment of the pathogen and improve the prognosis of patients. Furthermore, practitioners can administer targeted drugs to improve mitochondrial damage based on the levels of SCMM-CD4 in patients. Multidimensional treatment can be provided to patients with severe sepsis to improve their prognosis.

## Data Availability Statement

The original contributions presented in the study are included in the article/supplementary material, further inquiries can be directed to the corresponding author/s.

## Ethics Statement

The studies involving human participants were reviewed and approved by Ethics Committee of Zhejiang Provincial People's Hospital. The patients/participants provided their written informed consent to participate in this study.

## Author Contributions

QL, L-XP, and W-WC: study conception and design. LC, JF, C-XX, J-YL, and L-XP: material preparation, data collection, and analysis. L-XP: wrote the first draft of the manuscript. All authors contributed to the article and approved the submitted version.

## Funding

This study was supported by the Medical Health Science & Technology Program of the Zhejiang Provincial Health Commission (Nos. 2022KY530 and 2020KY438).

## Conflict of Interest

The authors declare that the research was conducted in the absence of any commercial or financial relationships that could be construed as a potential conflict of interest.

## Publisher's Note

All claims expressed in this article are solely those of the authors and do not necessarily represent those of their affiliated organizations, or those of the publisher, the editors and the reviewers. Any product that may be evaluated in this article, or claim that may be made by its manufacturer, is not guaranteed or endorsed by the publisher.
